# Measuring Mental Health Recovery: An Application of Rasch Modeling to the Consumer Recovery Measure

**DOI:** 10.1007/s11414-014-9411-1

**Published:** 2014-05-29

**Authors:** Kathryn KD Lusczakoski, P. Antonio Olmos-Gallo, William Milnor, Christopher J. McKinney

**Affiliations:** 1 Accelrys, Lafayette, CO USA; 2 Morgridge College of Education, University of Denver, Denver, CO USA; 3 Mental Health Center of Denver, Denver, CO USA; 4 Department of Psychology, University of Colorado at Denver, Denver, CO USA; 5 School of Psychological Sciences, University of Northern Colorado, Greeley, CO USA; 6 Research and Development, Mental Health Center of Denver, 4141 East Dickenson Place, Denver, CO 80222 USA

**Keywords:** Measurement, Recovery, Rasch Modeling, Classical Test Theory

## Abstract

As the need for recovery-oriented outcomes increases, it is critical to understand how numeric recovery scores are developed. In the current article, the modern Rasch modeling techniques were applied to establish numeric scores of consumers’ perceptions of recovery. A sample of 1,973 adult consumers at a community-based mental health center (57.5% male; average age of 47 years old) completed the 15-item Consumer Recovery Measure. A confirmatory factor analysis revealed the unidimensional nature of the Consumer Recovery Measure and provided construct validity evidence. The Rasch analysis displayed that the items produced acceptable model fit, reliability, and identified the difficulty of the items. The conclusion emphasizes the value of Rasch modeling regarding the measurement of recovery and its relevance to consumer-derived assessments in the clinical decision-making process.

## Introduction and Background

The concept of mental health recovery has received increased attention in the last decade as demonstrated by the multiple articles discussing qualitative^1^ and quantitative studies.^2^ Inherent to the process of understanding a theory or approach within a discipline is the development of psychometrically sound measurement tools to increase the understanding of the construct being measured. Thus far, several quantitative measures of consumers’ perceptions of their mental health recovery have been described in scientific journals, including: (a) illness, management, and recovery scale;^3^ (b) mental health recovery measure;^4^ (c) recovery assessment scale;^5^^,^ 6 (d) recovery-enhancing environment;^7^ (e) recovery knowledge inventory;^8^ (f) recovery process inventory;^9^ and (g) the stages of recovery instrument.^10^

All of these instruments have been based on a theory of measurement, known as Classical Test Theory (CTT), to determine their psychometric properties. The use of CTT to establish the psychometric properties of a recovery scale is congruent with the field of mental health research, where CTT has been, and still remains, the primary measurement theory of choice. However, in recent years, researchers in behavioral sciences have discovered the advantages of Item Response Theory (IRT), specifically the Rasch model, to expand the level of understanding gained in the measurement of psychological traits.^11^^–^ 14 This article describes the added value of the Rasch modeling focusing on the Consumer Recovery Measure (CRM), which was created by the Mental Health Center of Denver (MHCD) to measure consumer recovery, defined as “a non-linear process of growth by which people move from lower to higher levels of fulfillment in the areas of sense of safety, hope, symptom management, satisfaction with social networks and active/growth orientation”.^15^ The CRM was introduced as consumers’ views of their own self-recovery, as part of the holistic recovery evaluation system utilized internally by MHCD^16^. The goal of this article is to convey the overall essence of Rasch model to a wide variety of audiences, including clinical staff and researchers alike. The article is not intended as a technical or procedural guide to measurement theory, nor is it based in the methods of a particular software program. Due to the introductory nature of the article, several statistical and technical aspects of Rasch modeling are not discussed and/or presented. Researchers interested in the statistical underpinnings and procedural guides of Rasch modeling and IRT are invited to read more comprehensive descriptions in Bond and Fox,^17^ Embretson and Reise,^18^ and Hambleton et al.^19^ among others.

Mental health researchers, clinicians, administrators, and staff of the state and federal government have wrestled with the idea of how to measure mental health recovery.^20^^,^ 21 A familiar approach to measurement in mental health has been the development of quantitative surveys that can create a numeric score, which is hypothesized to be related to a consumer’s mental health recovery. When a numeric score is developed from a recovery survey, there are two critical issues: (1) How do we know the recovery survey will score consumers consistently (i.e., referred to as reliability)? and (2) How do we know the survey is truly measuring recovery and not something else (i.e., referred to as validity)? To answer these questions, survey developers use statistics to describe reliability (e.g., in CTT, Cronbach’s alpha, stability coefficients; in Rasch, person and item reliability) and validity estimates (e.g., concurrent and construct validity). These estimates are represented as mathematical models; however, rarely do mental health service providers conceptualize survey analysis in this manner. The following describes the benefits of using Rasch modeling to estimate the psychometric properties of recovery surveys.

Outside of the field of mental health recovery, psychological researchers increasingly are applying Rasch modeling techniques,^22^ as well as incorporating into their study conclusions the additional information provided by the Rasch measurement model.^23^ Rasch modeling is designed for instruments which are unidimensional (e.g., measure only one trait at a time; measure only recovery) with items that increase in a hierarchical order of difficulty.^18^ The hierarchical order of difficulty of the items in a survey is easier to understand when one thinks of a math test. For example, a hard question might be “What is the square root of 4,367?” and an easy question might be “What is 2 times 2?” Both questions measure math ability, but it can be assumed that someone who can answer the square root problem should have a higher math score than someone who can only answer the multiplication question. In terms of mental health recovery, some items may be more difficult (i.e., harder to agree with) than others. For example, on a four-point Likert-type scale, which ranges from strongly disagree = 1 to strongly agree = 4, a consumer who agrees with the question, “I have support during hard times,” would receive three units in a recovery score. If the same consumer agrees to another question in a different area of recovery (i.e., I am in control of my life), he/she also receives a recovery score of three units. The agreement to these two statements produces, numerically speaking, the exact same level of recovery; however, it is debatable if these two statements involve the same amount of recovery. Therefore, one might argue that consumers who feel that they are in control of their life may have a higher level of recovery than those who agree that they have support during hard times. Consequently, if consumers agree with a more difficult item, they should receive a higher recovery score (i.e., an ability score), than those who agree to a less difficult item, and Rasch modeling allows for these varying levels of item difficulty. In summary, Rasch models assume that some items may be more difficult to agree with and thereby, may be a more appropriate psychometric analysis for measuring recovery compared to Classical Test Theory analysis

Another advantage of Rasch modeling is that item parameters (i.e., item difficulty) and participant’s ability (i.e., person scores) are invariant, thus allowing recovery scores to be independently estimated from the item difficulty.^19^ This item/person invariance in Rasch modeling allows comparison among multiple recovery measures (i.e., assuming the same operational definition) with one ruler, as opposed to CTT which creates a separate ruler for each survey developed. For example, by applying Rasch modeling techniques, a researcher could compare two recovery surveys using an item/person map: (a) one developed for inpatient consumers with severe and persistent mental illness (SPMI) and (b) one developed for a less severe outpatient population. The recovery items may be easier for a less severe outpatient population and harder for an inpatient SPMI population. The comparison of various recovery surveys is an extreme benefit for any organization, which would receive recovery information via multiple surveys (e.g., state mental health centers, funding agencies, etc.).

Furthermore, the invariance of the item difficulty and person recovery scores is beneficial to the survey development process, allowing a survey developer to eliminate items that measure the same level of difficulty (e.g., the same level of recovery). Therefore, surveys developed with Rasch modeling techniques typically result in shorter surveys with high reliability.^17^ For example, the CRM began as a 100-item survey and was reduce to 15 items by removing questions that measured the same level of recovery difficulty, provided limited information (high error), and/or did not improve the overall survey reliability estimates. The reduction of items allowed for the development of a final 15-item scale that included monotonically increasing items to measure self-perception of recovery. Furthermore, Rasch analysis is extremely beneficial for computer-adaptive testing scenarios^24^, which is an ideal future direction, but are rarely found in mental health services. The current article only addresses the psychometrics of current version of the CRM (version 3.0) and does not describe details of the development process. Interested parties should contact the authors for more information on the development process of the CRM.

A further distinction of Rasch measurement is a specific focus on the measurement of latent traits which cannot be observed or measured directly. Latent traits are commonly encountered in psychopathology because mental health and associated cognitive concepts cannot be directly measured. Along with the assumption of a latent trait, there are two additional assumptions—local independence and unidimensionality. Local independence requires that, when the consumer’s recovery ability is held constant, the consumer’s responses to each item are independent of each other. Unidimensionality of the latent trait implies that there is only one trait being measured by the instrument.^25^ Many definitions of mental health recovery include the terms multifaceted or multidimensional, suggesting that mental health recovery includes multiple constructs interacting at various levels.^2^ It should be noted that, in the MHCD measurement model of mental health recovery, the CRM is one of three measures of mental health recovery. Therefore, the CRM is designed to measure the unidimensional latent trait of consumers’ perceptions of their own recovery, while being in agreement with MHCD’s theoretical definition of recovery.

In summary, Rasch modeling provides the following advantages: (1) allows for varying item difficulty, meaning that some items may be more difficult to agree with; (2) allows for comparisons among multiple recovery measures on one ruler; (3) develops highly reliable surveys with fewer items; and (4) is designed to measure latent traits. The aim of this study is to illustrate the application of Rasch modeling techniques to measure mental health recovery using the CRM.

## Participants

The sample consisted of 1,973 adult consumers receiving mental health services at MHCD from May 2005 to August 2007, and excluded consumers who received services in nursing home facilities. The sample of consumers was 57.5% male and 42.5% female, with an age range of 21–80 years, and an average of 47 years. The self-reported race/ethnicity of the consumer participants included 55.6% non-Latino white, 25.3% African American, 13.0% Latino, 3.6% multiracial, 1.1% American Indian, 0.8% Asian, and 0.6% Hawaiian-Pacific Islander. The primary diagnoses of the consumers, according to the DSM-Axis I^26^, were (a) 29.5% schizophrenia, (b) 25.5% bipolar, (c) 21.8% schizoaffective, (d) 11.7% major depression, (e) 4.6% other psychotic disabilities, (f) 3.6% anxiety, (g) 1.9% other nonpsychotic disorders, (h) 0.6% dysthymia, (i) 0.4% personality impulse, and (j) 0.4% delusion. In addition, 48.6% of the consumers reported a co-occurring substance abuse diagnosis.

## Measure

The CRM is a measure of consumers’ perception of their own mental health recovery, developed by the Recovery Committee at the MHCD in 2003. The Recovery Committee was established at MHCD in 2000 and consists of consumers, managers, and staff members. More detail on the recovery committee can be found in the Olmos-Gallo et al.’s article.^16^ The CRM was developed through several steps that included the development of a logic model, literature reviews, followed by focus groups conducted with consumers at MHCD, and continuous revisions by the Recovery Committee. The current version of the CRM (version 3.0) consists of 15 items related to active/growth orientation, hope, symptom interference, safety, and social network, with four potential response sets that range from strongly agree (4) to strongly disagree (1). Three items are reverse coded. The CRM is a self-report instrument completed in a web-based or scannable form by consumers every 3 months. Table 1 provides the text of each question on the CRM.

**Table 1:** Text of 15 items on CRM

Item	Text of item	Item category
Q1	Lately I feel I have been making important contributions	Active growth
Q2	I have hope for the future	Hope
Q3	I am reaching my goals	Active growth
Q4	I have this feeling things are going to be just fine	Hope
Q5	Recently my life has felt meaningful	Hope
Q6	Recently I have been motivated to try new things	Active growth
Q7	I get a lot of support during the hard times	Social network
Q8	In most situations I feel totally safe	Safety
R-Q9	My life is often disrupted by my symptoms	Symptom management
R-Q10	Sometimes I am afraid someone might hurt me	Safety
Q11	I have people in my life I can really count on	Social network
R-Q12	Life's pressures lead me to lose control	Symptom management
Q13	I have friends or family I really like	Social network
Q14	My symptoms interfere less and less with my life	Symptom management
Q15	When my symptoms occur, I am able to manage them without falling apart	Symptom management

The reference item number, item text, and general item category is provided. Reverse-coded items are denoted with an R preceding the reference item number

## Data Analysis

The sample data were transferred from MHCD’s Management Information System to SPSS^27^ and then exported to WINSTEPS^28^ for Rasch analyses and LISREL^29^ to provide construct validity evidence and support for the unidimensional structure assumption of Rasch modeling. For the Rasch analysis, the specific model implemented was the Rasch Rating Scale Model because it allows Likert-type response sets, similar to those found in the CRM.^30^ To provide construct validity evidence, a confirmatory factor analysis (CFA) was completed to validate the theorized unidimensional structure of the CRM using robust maximum likelihood estimation. The authors utilized fit indices cutoff values based on standard CFA criteria: a nonsignificant chi-square test, RMSEA ≤0.08, NNFI >0.90, and CFI >0.90.^25^ This study was approved by the members of the University of Northern Colorado Institution Review Board.

## Construct Validity Evidence: Confirmatory Factor Analysis

**Figure 1:**
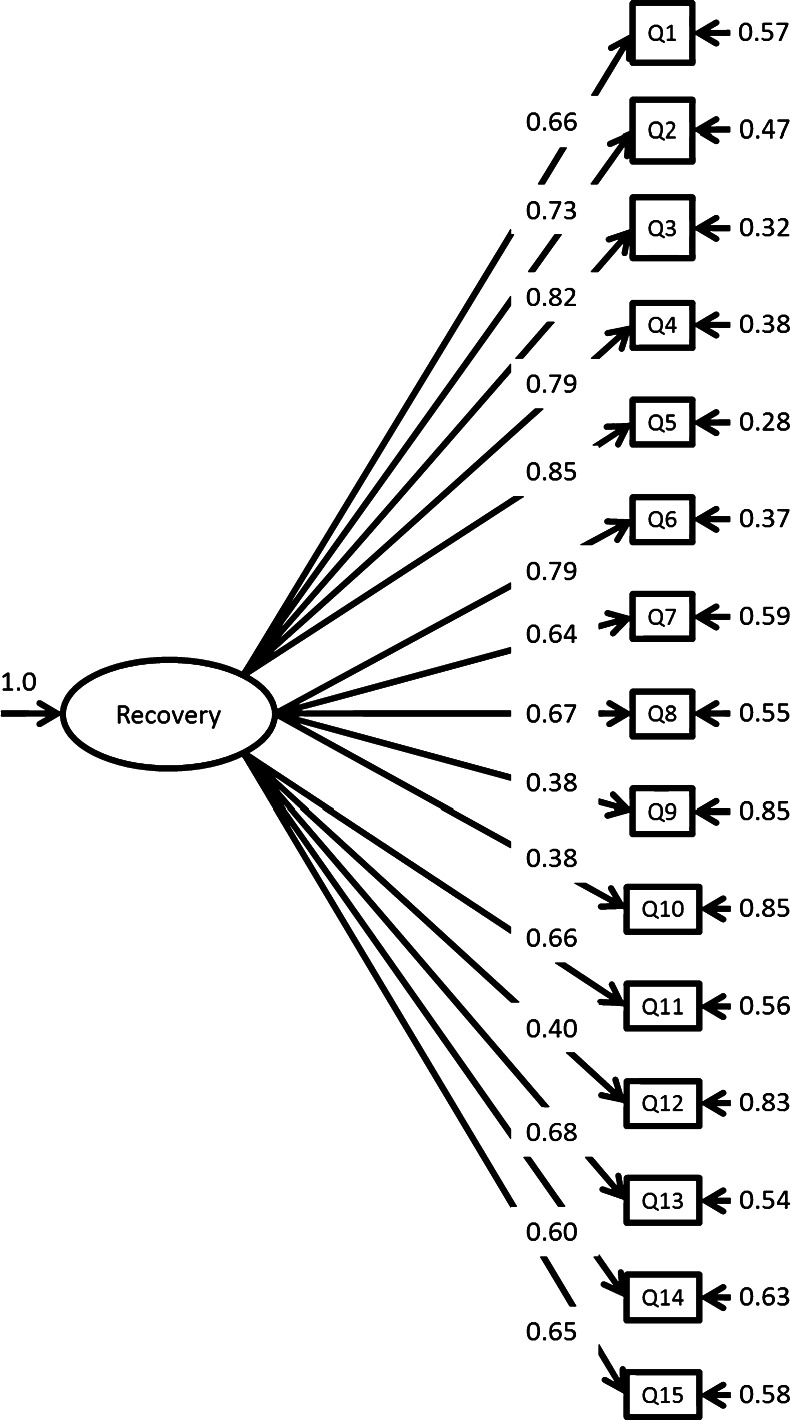
The Consumer Recovery Measures unidimensional structure with standardized loadings

Prior to conducting a Rasch analysis, a confirmatory factor analysis was conducted to examine the unidimensionality and construct validity of the CRM survey. The sample of 1,973 consumers was divided into a calibration sample (*N* = 987) and a validation sample (*N* = 986). Using the calibration group, the loadings for the structures were estimated. These loadings were then fixed and compared against the validation sample. The unidimensional structure displayed acceptable fit indices and was assumed to have adequate model fit; RMSEA = 0.078, CFI = 0.97, NFI = 0.97, NNFI = 0.97, Satorra-Bentler *χ* ^*2*^(*df* = 90) = 630.3, *p* < 0.05. Acceptable model fit evidence was also shown for the validation sample; RMSEA = 0.095, CFI = 0.95, NFI = 0.95, NNFI = 0.96, Satorra-Bentler *χ* ^*2*^(*df* = 120) = 1,187.8, *p* < 0.05. Figure 1 displays a diagram of the unidimensional structure with estimated standardized loadings. All item loading values were found to be statistically significant, at the 95% confidence level, regardless of magnitude. Both second- and high-order CFA models were examined and found to produce less than ideal model fit, compared to the unidimensional model. Therefore, the CFA results provided construct validity evidence and support for the unidimensional structure of the CRM.

## Rasch Analysis: Item Fit/Person Fit and Reliability Estimates

Rasch modeling techniques typically assess model fit in two ways: (1) how well the items fit the recovery ruler (i.e., item-fit) and (2) how well the consumers fit the recovery ruler (i.e., person-fit). Two common fit indices reported in Rasch modeling include mean square infit and outfit estimates, indicating how well the recovery items (response patterns) fit a standard Rasch, logistic-based, mathematical model. In general statistical terms, the infit and outfit estimates are two fit indices that summarize the squared standardized residual of the response options. A detailed discussion of these indices is beyond the scope of this article; however, interested parties should reference Bond and Fox.^17^ The infit and outfit estimates are computed for every item and every consumer to indicate how well they fit the measurement model (i.e., match the recovery ruler). Both infit and outfit estimates are always positive with 1.0 indicating perfect fit, and values range from 0.60 to 1.40 suggesting adequate model fit.^17^ All items produced acceptable infit and outfit statistics excluding question 10 (i.e., infit = 1.44, outfit = 1.60), which remained in the analysis to ensure that the hypothesized concept of recovery was properly defined. The overall item (i.e., infit = 1.00, outfit = 1.04) and person (i.e., infit = 1.05, outfit = 1.05) fit indices were acceptable. Table 2 provides a complete list of the item fit statistics for each of the CRM items. Reliability estimates in Rasch modeling are separated by person and item, but their interpretation is similar to Cronbach’s alpha, in the sense that scores range from 0 to 1; 1 indicates perfect reliability. The CRM reliability estimates are as follows: person = 0.83 and item = 0.99.

**Table 2:** Rasch fit statistics for 15-item CRM

	Infit	Outfit
Item fit statistics		
R-Q10	1.44	1.60
R-Q9	1.29	1.36
R-Q12	1.25	1.33
Q13	1.18	1.21
Q11	1.11	1.16
Q6	1.05	1.12
Q7	0.98	1.04
Q14	0.94	1.02
Q1	0.96	0.99
Q15	0.88	0.91
Q8	0.87	0.86
Q2	0.82	0.80
Q3	0.77	0.78
Q4	0.76	0.76
Q5	0.72	0.73
Total	1.00	1.04
Person fit statistics		
Total	1.05	1.05

All individual person fit statistics were computed but are not displayed

## Rasch Analysis: Item Difficulty

**Figure 2:**
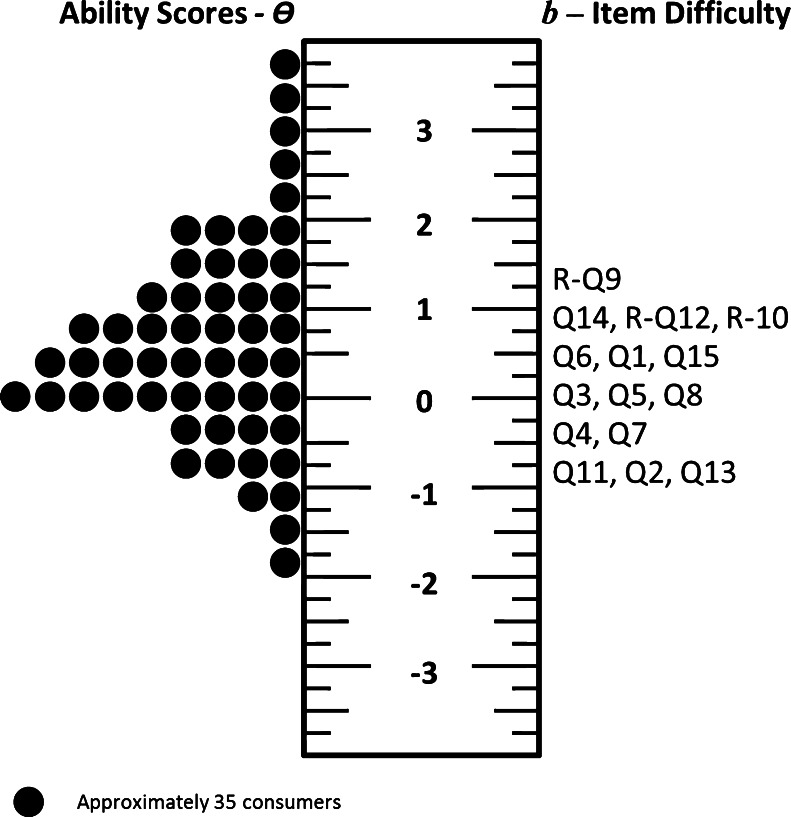
The item/person map of the Consumer Recovery Measures. The distribution of consumers’ ability scores is shown on the left side of the ruler; difficulty scores are depicted on the right side of the ruler

As described before, a key advantage of Rasch modeling is that the item/person invariance allows for the creation of an item/person map, which displays the distribution of consumer recovery scores (i.e., denoted as θ) on the same scale as the distribution of average item difficulty values (i.e., denoted as *b*). Most of the consumer recovery scores were distributed around a mean of 0.73 (SD = 1.25), and the average item difficulty scores was zero but less variation (SD = 0.44). It is important to notice that the item/person map can provide insight into the construct validity of the CRM by showing that items are at the appropriate difficulty level compared to the consumer ability scores. In this regard, the mean and range of Likert item scores are distributed around the same level as the item difficulties, but with the consumer’s recovery score being slightly higher than the item difficulty. Figure 2 shows the item/person map for the CRM scores and item difficulty scores.

By examining the difficulty of the items, important information about the concept of recovery was gained. For example, the hardest item to endorse is Q9, life not being disrupted by symptoms (*b* = 0.64). Also, other symptom-related items Q14 (*b* = 0.45) and Q12 (*b* = 0.49) have high average difficulty scores. On the other hand, Q15, “I am able to manage my symptoms without falling apart” has a lower average difficulty score (*b* = 0.13). A potential explanation is that consumers with an above-average recovery score may still have symptom interference in their daily lives. However, for those same consumers, the ability to manage symptoms without falling apart is easier to achieve. In addition, questions related to social networks and hope provide some interesting item difficulty information. The analysis showed that the items related to social networks are three of the four easiest items (*b* = −0.75, −0.46, and −0.24). Question 13 discusses having friends and family you like, while Q7 discusses getting support during the hard times. However, Q7 is much easier to endorse by approximately a half standard deviation. Regarding hope, Q2 (*b* = −0.68), which discussed having hope for the future is the second easiest item. However, the two other hope questions (i.e., Q4 and Q5, both related to the idea that things are going to be fine) are just below the average difficulty score. Table 3 provides the complete list of Rasch difficulty scores for all 15 CRM items.

**Table 3:** Rasch difficulty scores for each item and potential response

Question	Difficulty scores					Type of questions
	Average (*b*)	Average for each potential response				
		Strongly agree	Agree	Disagree	Strongly disagree	
R-Q9	1.01	0.01	0.24	0.68	1.62	Symptom interference
Q14	0.45	−0.09	0.48	1.06	2.07	Symptom interference
R-Q12	0.39	−0.13	0.23	0.74	1.94	Symptom interference
R-Q10	0.25	−0.42	−0.22	0.32	1.34	Safety
Q6	0.16	−0.37	−0.19	0.40	1.52	Active growth
Q1	0.15	−0.59	0.20	0.70	1.82	Active growth
Q15	0.13	−0.62	−0.10	0.52	1.67	Symptom interference
Q3	−0.01	−0.43	0.14	0.79	2.13	Active growth
Q5	−0.05	−0.70	0.05	0.74	1.94	Hope
Q8	−0.09	−0.60	−0.07	0.66	2.03	Safety
Q4	−0.24	−0.86	−0.08	0.63	1.89	Hope
Q7	−0.24	−1.07	−0.49	0.37	1.57	Social network
Q11	−0.46	−0.92	−0.13	0.67	1.99	Social network
Q2	−0.68	−0.96	−0.27	0.56	1.92	Hope
Q13	−0.75	−0.85	−0.21	0.61	1.99	Social network
Average	0	−0.57	−0.02	0.63	1.83	

## Rasch Analysis: Unique Response Patterns

One more advantage of Rasch modeling is its ability to investigate why some consumers may not answer in the order of difficulty determined by the majority of the population. Investigations into unexpected patterns of response (i.e., misfitting responses) may further increase our understanding of the unique path toward recovery. As part of this analysis, consumers with the most frequent misfitting patterns were reviewed. They included unexpected high scores for Q15 and Q9 (i.e., both regarding symptom management) and low scores for Q13 (i.e., social network). This seems to suggest that some consumers may be able to manage their symptoms even when they may not be doing well in other areas. Regarding Q13, we hypothesize that it may be related to the language in the question (i.e., having friends/family the consumer really likes). Perhaps consumers feel they have support regardless of whether or not they like those persons within their social network.

## Discussion

The purpose of this paper was twofold: (1) to introduce the CRM as a reliable and valid instrument for the unidimensional measure of consumers’ perceptions of their own mental health recovery and (2) to display an application of Rasch psychometric analysis to a recovery measure. Regarding the second point, Rasch demonstrates value added in at least four areas. First, the process of recovery may correspond to the assumptions of Rasch modeling (e.g., all items are of equal difficulty), as discussed in the introduction. Second, Rasch modeling provided practical information for clinical decision making. Third, Rasch modeling can separate out item difficulty and participant’s score, in that, it provides a deeper understanding of how some concepts related to recovery are more difficult to endorse than others. Fourth, Rasch modeling allowed for the examination of unique response patterns, which increase the understanding of individualized change in mental health recovery. The results suggest that the field of adult mental health recovery could greatly benefit from the unique information provided by Rasch modeling.

Rasch analysis allows for an in-depth investigation into the concept of consumers’ perceptions of mental health recovery, with implications for recovery-oriented clinical practices and research. For example, the Rasch analysis suggested that one of our symptom interference items (i.e., about managing symptoms) is related to a moderate recovery level; while another (i.e., life not being disrupted by symptoms) is related to the highest recovery level. This seems to indicate that consumers perceive a big difference between simply experiencing symptoms versus feeling that they are able to manage their symptoms without falling apart. Previous researchers^2^ have emphasized that the experience of symptoms is not a critical component of recovery; however, consumers’ ability to cope, manage, and live fulfilling lives, in light of their symptoms, are more central concepts in recovery. Therefore, consumers may notice improvements in several areas like hope, social relationships, active growth, and sense of safety but still experience, and/or struggle with, symptoms.

Three of the four easiest items on the CRM were related to social networks. A review of the questions showed that easier-to-endorse items were related to having friends and family they like, while getting support during the hard times had an average difficulty level. Therefore, consumers in lower levels of recovery may have social networks, but they may not feel fully supported during the hard times. The second easiest item was related to having hope for the future. It should be noted that the participants involved in this sample were receiving mental health treatment. From a clinical point of view, it seems paramount that consumers will show some hope for the future if they are willing to voluntarily seek treatment. Upon entry into treatment, typically, clinicians try to establish social networks and increase the hope of the consumers; therefore, it is understandable why these are the easiest items.

Also, the Rasch analysis of the CRM suggested that the unique response patterns were due to consumers with unexpected high scores in symptom management and unexpected low scores in social networks. It has been observed that, sometimes, consumers manage their symptoms, despite the fact that they were not doing well in other, perhaps, easier areas. The findings support the notion that consumers can have a large variability in how they manage their symptoms during the recovery process. The unexpected low scores related to having friends and family, which the consumers really like, may be related to the language in the questions (i.e., really like). Consumers may have friends and family, and they may have support systems; however, they may not necessarily really like their family. As a result, consumers may score high in other areas such as hope, active growth, safety, and symptom interference, and not feel that they have someone they really like. The item was reviewed by the Recovery committee and the decision was made to leave it in the scale and review it in future validation studies.

In summary, the Rasch psychometric validation provided preliminary support for the CRM as a valid and reliable unidimensional measure of consumers’ self-perceptions of mental health recovery as confirmed through confirmatory factor analysis and item response theory. We do not disregard traditional psychometric procedures (e.g., CTT) as a suitable method for scale development and scoring. Nevertheless, Rasch measurement may be more congruent to the theory of mental health recovery and will provide additional information with clinical relevance. In spite of these benefits, Rasch modeling is more mathematically complex and is only beginning to emerge as standard training in social science graduate programs. In recent years, Rasch modeling trainings have been provided more frequently (e.g., American Psychological Association, American Evaluation Association), and software has become more user friendly, with professional graphic programs to assist in interpretation (e.g., Winsteps).

## Limitations

Due to the introductory nature of this article, various constructs related to the mathematical underpinnings of Rasch modeling were not discussed and should be reviewed before determining the most appropriate model to measure the trait of interest. Furthermore, the results need to be verified through qualitative techniques with consumers and clinicians, along with examination of additional samples of consumers to increase the validity of the findings. The sample used for validation in this study consisted of adults with severe and persistent mental illness, as defined by the State Mental Health Authority. Therefore, the sample can be considered representative of individuals with a major mental illness, receiving mental health services from community mental health centers in major metropolitan areas in the USA. However, they may not represent other populations with mental illness, or receiving mental health services from other sources, such as private providers and mental health services provided through jails or mental health hospitals. Lastly, despite the fact that Rasch methods provide initial validation evidence, specifically regarding construct validity, full validation requires comparisons to other outcome measures and statistical procedures. The authors are currently in the process of conducting these additional validation procedures.

## Implications for Behavioral Health

The mental health field is moving toward more accountability and fiscal responsibility with State and Federal regulations requiring the use of evidence-based programs. Internally, outcomes are frequently used for quality improvement and clinical decision making. Within this context of increased accountability, the measurement of recovery is relatively new and, in practice, it competes with the long-standing, dominant medical model of assessment and treatment. On the practical side, nearly all reimbursements are driven by defined and approved service interventions, resulting in many organizations using billable hours as a measure of performance and productivity. Although the use of billable hours as an indicator of client outcomes can be appropriate, we have found an even stronger relationship between service delivery and outcomes as measured by the Consumer Recovery Measure. Therefore, the use of a recovery-based measurement has allowed our center to move past the traditional symptom management model based on service hours, and provide the clinical staff with specific indicators that can be targeted through strengths-based service delivery approaches. The organizational challenge is to adopt, support, and reinforce tools that clearly inform clinical action and result in a benefit to the consumers. As described in the current study, Rasch modeling may provide additional insight regarding mental health recovery, as well as a more informative analysis of consumers’ perception about their recovery. It is our hope that the benefits of using Rasch modeling for mental health recovery will move the field forward and demonstrate the relevance of service delivery that matches the expected outcomes.
